# Variation in expenditure for common, high cost surgical procedures in a working age population: implications for reimbursement reform

**DOI:** 10.1186/s12913-019-4729-2

**Published:** 2019-11-21

**Authors:** W. Wynn-Jones, T. P. Koehlmoos, C. Tompkins, A. Navathe, S. Lipsitz, N. K. Kwon, P. A. Learn, C. Madsen, A. Schoenfeld, J. S. Weissman

**Affiliations:** 1 Centre for Surgery and Public Health, Brigham and Women’s Hospital, 1620 Tremont Street, 1 Brigham Circle, Boston, MA 02120 USA; 2 F. Edward Hebert School of Medicine, Uniformed Services University of the Health Sciences, 4301 Jones Bridge Road, Bethesda, MD 20184 USA; 3 Heller Graduate School, Brandeis University, 415 South St., Waltham, MA 02354 USA; 4 Perelman School of Medicine, University of Pennsylvania, Philadelphia, PA USA; 5 Division of General Internal Medicine and Center for Surgery and Public Health, Brigham and Women’s Hospital and Harvard Medical School, Boston, USA; 6 Centre for Surgery and Public Health, Brigham and Women’s Hospital, Boston, USA; 7 Department of Surgery, F. Edward Hebert School of Medicine, Uniformed Services University of the Health Sciences, 4301 Jones Bridge Road, Bethesda, MD 20814 USA; 8 Henry M Jackson Foundation for the Advancement of Military Medicine, Inc., Bethesda, MD USA; 9 Department of Orthopaedic Surgery Center for Surgery and Public health Brigham and Women’s Hospital Harvard Medical School, Boston, USA; 10 (Health Policy) Harvard Medical School, Center for Surgery and Public Health, Boston, USA

**Keywords:** Bundled payments, Military medicine, TRICARE, CABG, Colectomy, Lumbar spinal fusion, Total knee replacement, Total hip replacement, Value-based care

## Abstract

**Background:**

In the move toward value-based care, bundled payments are believed to reduce waste and improve coordination. Some commercial insurers have addressed this through the use of bundled payment, the provision of one fee for all care associated with a given index procedure. This system was pioneered by Medicare, using a population generally over 65 years of age, and despite its adoption by mainstream insurers, little is known of bundled payments’ ability to reduce variation or cost in a working-age population. This study uses a universally-insured, nationally-representative population of adults aged 18–65 to examine the effect of bundled payments for five high-cost surgical procedures which are known to vary widely in Medicare reimbursement: hip replacement, knee replacement, coronary artery bypass grafting (CABG), lumbar spinal fusion, and colectomy.

**Methods:**

Five procedures conducted on adults aged 18–65 were identified from the TRICARE database from 2011 to 2014. A 90-day period from index procedure was used to determine episodes of associated post-acute care. Data was sorted by Zip code into hospital referral regions (HRR). Payments were determined from TRICARE reimbursement records, they were subsequently price standardized and adjusted for patient and surgical characteristics. Variation was assessed by stratifying the HRR into quintiles by spending for each index procedure.

**Results:**

After adjusting for case mix, significant inter-quintile variation was observed for all procedures, with knee replacement showing the greatest variation in both index surgery (107%) and total cost of care (75%). Readmission was a driver of variation for colectomy and CABG, with absolute cost variation of $17,257 and $13,289 respectively. Other post-acute care spending was low overall (≤$1606, for CABG).

**Conclusions:**

This study demonstrates significant regional variation in total spending for these procedures, but much lower spending for post-acute care than previously demonstrated by similar procedures in Medicare. Targeting post-acute care spending, a common approach taken by providers in bundled payment arrangements with Medicare, may be less fruitful in working aged populations.

## Background

The United States is well-established as having the highest per capita expenditure on healthcare without corresponding improvement in overall health, due in part to fragmentation of care services and cost variability between regions and providers [1, 2] .

Various methods of payment reform are suggested to control costs, efficiency, and variability, including bundled payments as used by Medicare. These are delivered as a fixed payment amount to a single provider entity and intended to cover the full range of services for an episode of care. Studies in Medicare [3, 4] have established that much of the variation occurs not only during the index admission (e.g., due to differential use of ICUs or long lengths of stay), but also after discharge from hospital, including readmissions and use of post-acute services. This is in line with current studies showing regional variation in hospital cost, which in turn is generally driven by clinical practice rather than patient characteristics [2].

Investigations by the Centers for Medicare and Medicaid Services (CMS) have identified surgery as an attractive target for payment reform using bundling, due to the large number of inpatient surgical episodes covered by Medicare [5], and the high variability in related expenditures [6, 7]. However, this foundational work has not been performed for non-Medicare populations, even though commercial insurers have begun using similar models in their working-age populations. Therefore, identifying similar patterns of variation in younger populations is critical.

This study addresses the gap by examining TRICARE data to evaluate baseline variation in expenditures for five common surgical procedures-- primary hip and knee replacement (PHR and PKR), coronary artery bypass graft (CABG), spinal fusion and colectomy [6, 7],--to discern their suitability for bundled payments in a working-age population.

The TRICARE population is nationally and demographically representative of adults from 18 to 65 [8], and the reimbursement schedule for purchased care follows that of Medicare, making this system a useful model for studying bundled payments in the care of working-age adults. Results are expected to inform discussion for use of bundled payment models to reduce surgical cost variability in working-age populations.

## Methods

### Study data and methods

TRICARE is the primary insurance product for US military personnel and their families, covering 9.5 million beneficiaries, approximately 80% of whom are not active-duty [8]. The system is bifurcated, with services provided either in direct care (at military facilities) or purchased care (in the civilian sector). This study examined TRICARE Prime claims data for all patients undergoing selected inpatient procedures between October 1, 2011 and September 30, 2014. This three-year timeline was selected based on a previous TRICARE bundled payment trial in a regional market [9].Patients undergoing primary elective hip replacement (PHR), primary elective knee replacement (PKR), primary coronary artery bypass grafting (CABG), lumbar spinal fusions (LSF), and colectomy were identified from purchased care TRICARE claims based on the presence of the corresponding procedure codes from the International Classification of Diseases ICD, Ninth Revision [4, 5] (see Additional files 1, 2, 3 and 4). These procedures were selected because they vary widely in Medicare reimbursement [6, 7], are sensitive to bundling-based reduction in cost [10], and are known to occur frequently in working-age adults [11–15]. Additionally, these procedures are associated with high costs and have been used as measures of surgical healthcare quality in prior research [16–19]. The study excluded operations performed due to trauma for PHR and PKR, and LSF operations with evidence of concomitant fusion surgery at a different spinal level within their reference inpatient claim. Generally only elective surgery was considered in all cases.

To minimize episodes of care with incomplete information, patients who had evidence of dual coverage by another payer were also excluded from the analysis (20%). Patients admitted for surgery from either a long-term care facility, skilled nursing facility or a hospice [6] (< 1%), or who died before discharge following the index surgery [6] (< 1%) were similarly excluded in order to focus on variation in a more homogeneous patient cohort. Demographic information was used in case-mix adjustment, including age, sex, race and sponsor’s military rank. Sponsor’s rank has previously been used as a proxy for socioeconomic status in studies using TRICARE, with junior enlisted sponsor rank considered representative of low socio-economic status [20, 21]. In the case of missing race data, the sponsor’s race was used [21].

### Calculation of TRICARE payments

For this study, payments made by TRICARE on behalf of each patient to commercial healthcare providers were used rather than submitted charges by the hospitals [5]. Payment information was extracted for all services made on behalf of each patient within the study period, including inpatient and outpatient, from six months prior to the date of admission for one of the index procedures, through 90 days after the discharge date. We used 90 days as this is the length of time commonly employed by CMS in its Bundled Payment model design [22, 23]. All patient specific payment information for the six months prior to the index admission was aggregated and used in later case mix adjustment of episodes.

All payments made for care delivered during the index procedure and the 90-day period after the discharge from the index procedure were subsequently sorted into four discrete components: Index hospitalization; Readmission; Post-acute care; and Healthcare Professional Fees. The Index hospitalization payment was the total amount paid by TRICARE for the reference inpatient procedure [6, 7]. Readmission payments were considered for any inpatient procedure occurring after discharge from the index hospitalization but within the 90-day period. Included in the Post-acute care category were payments for acute or long-stay rehabilitation hospital, skilled nursing facility, outpatient services, home health care, hospice, durable medical equipment, as well as facility fees for outpatient care. Healthcare professional fees were included in a separate category.

### Statistical analysis

For each surgery type, all discrete episodes within an eligible time period were grouped using the provider zip code into Hospital Referral Regions (HRR) [24]. Our Data Use Agreement with TRICARE prevented us from identifying individual hospitals within the dataset, so only provider zip codes were available. Since provider zip codes did not provide sufficient granularity for hospital level analysis, we consequently chose HRRs as the most appropriate aggregate unit of analysis. HRRs with fewer than five cases during the study period were excluded from further analysis, representing 7% (225) of episodes for PHR, 3% (186) of episodes for PKR, 15% (197) of episodes for CABG, 3% (201) of episodes for LSF, and 42% (688) of episodes for colectomy.

To account for regional differences in TRICARE payments, price standardization was applied to all payments following methods outlined in similar Medicare analyses [25, 26], adapted to the claims structure within TRICARE. Geographic spending variation due to wage indices was consequently removed with this method.

Case mix adjustments were made for patients’ age, sex, race, and sponsor’s military rank; admission acuity; and procedural resource use intensity for CABG, colectomy and lumbar spinal fusion surgeries. In the specific case of colectomy, adjustments were made for overall management of cancer versus non-cancer since these were deemed to be distinct types of cases with considerable differences in their post-acute care pathways. To account for comorbidities, we included the Charlson index for each patient. Following the precedent set in similar Medicare analyses we also adjusted for total prior six months’ healthcare payments made by TRICARE on behalf of the patient. These case-mix adjustments were implemented using generalized linear mixed models, using a log link with a random effect for HRR (which accounts for clustering of outcomes from patients within an HRR). The random effect was assumed to have a gamma distribution, which has been found in the literature [27] to be appropriate for modelling cost data. From the generalized linear mixed model, for each HRR we obtained one case-mix adjusted, empirical Bayes reliability-adjusted [28] estimate of the mean cost for the HRR. HRRs were then initially ranked according to reliability-adjusted total payments made by definitive surgery type for 90 days of care subsequent to the index admission. Quintiles were then formed and the average per quintile reported with confidence intervals. HRRs within each of the definitive surgery types were also separately reranked for index hospitalization payments, Readmission payments, Post-acute care payments, and Healthcare Professional fees. New quintiles were formed, and averages and confidence intervals were calculated.

All analyses were performed using SAS version 9.4 and a *P*-value less than 0.05 was considered statistically significant. This work was found exempt by the Institutional Review Board of the Uniformed Services University of the Health Sciences and Partners Institutional Review Board.

## Results

Following identification of index episodes of interest our pre-exclusion cohort consisted of: 6473 PHR; 12,291 PKR, 2872 CABG; 20,187 LSF, and 2368 colectomy. The total number of episodes after the exclusions outlined in the methods were 21,346. A total of 197 HRRs across the nation performed these services, with 129 HRRs performing PHR, 177 performing PKR, 86 performing CABG, 155 performing LSF, and 76 performing colectomy. The total average payments made by TRICARE on behalf of its beneficiaries for all care provided within a 90-day period were: $18,566 (PHR), $19,782 (PKR), $44,983 (CABG), $34,554 (LSF), and $29,376 (colectomy). Substantial variation was observed in the total payments for each procedure type across the HRRs after price standardization (Table 1). The largest inter-quintile variation (measured by percentage difference) was observed in Colectomy surgery with average total payments in the most expensive HRR quintile being 154% more than in the least expensive quintile, $46,939 vs $18,459 (also $28,480 absolute difference.) The percentage increases from lowest to highest quintile for each procedure were 101% (PHR), 89% (PKR), 105% (CABG), and 90% (LSF) respectively. Case mix adjustment had the effect of reducing the observed variation within the total payments by procedure type. After price standardization and case mix adjustment, the highest variation in total payment was observed in PKR (75%), while the variation for other procedures was 74% in PHR, 73% in LSF, 66% in CABG and 64% in colectomy.

**Table 1:** Price standardized (first line) and fully adjusted (second line) average total TRICARE purchased care payments around episodes of five common inpatient procedures, 2012–2014. Difference reflects the variation between highest and lowest quintiles, shown as a dollar value and as a percentage of the lowest quintile value

		1 (lowest)	2	3	4	5 (highest)	Difference (% of 1st quintile)
PHR	Price standard	$14,228	$15,796	$16,989	$18,706	$28,613	$14,385 (101%)
	+ case-mix	$15,039	$16,042	$17,049	$18,486	$26,216	$11,177 (74%)
PKR	Price standard	$15,562	$16,851	$18,151	$19,718	$29,512	$13,950 (90%)
	+ case-mix	$16,046	$17,111	$18,157	$19,559	$28,037	$11,990 (75%)
CABG	Price standard	$33,205	$38,170	$43,085	$49,830	$68,076	$34,870 (105%)
Surgery	+ case-mix	$35,801	$39,256	$42,980	$47,518	$59,360	$23,558 (66%)
Lumbar	Price standard	$26,253	$29,777	$32,641	$35,928	$49,961	$23,707 (90%)
Spinal Fusion	+ case-mix	$27,271	$30,337	$32,694	$35,256	$47,210	$19,939 (73%)
Colectomy	Price standard	$18,458	$23,940	$27,761	$33,850	$46,939	$28,480 (154%)
	+ case-mix	$23,152	$25,726	$28,340	$31,670	$37,994	$14,842 (64%)

We also determined the variation for each of the price standardized and case mix adjusted cost components by HRR for each of the discrete surgery types (Table 2). Index Hospitalization payments differed greatly between the quintiles across the HRRs for all of the procedure types with the greatest variation being observed in PKR (107%, $11,324). We separately considered the source of this variation in Index Hospitalization payments for CABG which exhibited the largest inter-quintile difference observed in our study. The heterogeneity of Diagnosis Related Group (DRG) payments within the procedural grouping explained some of the variation but variation in clinical practice is also likely to contribute to this variation. The inter-quintile differences for Healthcare Professional fees between the surgical procedure types across the HRRs was considerable, with the lowest variation observed in PHR (22%, $722) and the highest seen in colectomy and CABG (46%, $1014 and $1019 respectively). Total payments for post-acute care were low in this population, with average total payments of $628 for PHR, $989 for PKR, $1606 for CABG, $663 for LSF, and $1517 for colectomy.

**Table 2:** Adjusted average TRICARE purchased care payments for different components of care around surgical episodes, by procedure after price standardization and case-mix adjustment, 2012–2014. Difference reflects the variation between the highest and lowest quintiles

DRG	Cost	Quintile (Total payments, by HRR quintile ($))					Difference
		1 (Lowest)	2	3	4	5 (Highest)	
Colectomy	Index hosp.	13,466	$15,591	$17,509	$20,067	$27,141	$13,674
	Readmissions	$1626	$9949	$10,892	$12,722	$18,883	$17,257
	Phys. fees	$5019	$5457	$5814	$6371	$7320	$2301
	Post-acute	$1179	$1370	$1539	$1750	$2194	$1014
PKR	Index hosp.	$10,568	$11,434	$12,172	$13,393	$21,893	$11,324
	Readmissions	$0	$8449	$9645	$10,818	$14,278	$14,278
	Phys. fees	$3830	$4259	$4521	$4762	$5306	$1475
	Post-acute	$840	$952	$986	$1031	$1138	$298
PHR	Index hosp.	$10,588	$11,403	$12,165	$13,324	$21,611	$11,022
	Readmissions	$0	$10,793	$11,528	$12,262	$13,885	$13,885
	Phys. fees	$3303	$3494	$3597	$3767	$4026	$722
	Post-acute	$534	$593	$625	$656	$732	$197
Lumbar	Index hosp.	$18,872	$21,506	$22,994	$25,793	$37,705	$18,833
Spinal	Readmissions	$0	$6970	$8507	$10,082	$14,607	$14,607
Fusion	Phys. fees	$6463	$7343	$7882	$8418	$9746.	$3282
	Post-acute	$421	$550	$629	$743	$974	$553
CABG	Index hosp.	$26,339	$28,848	$32,877	$36,945	$48,867	$22,527
Surgery	Readmissions	$0	$7175	$8827	$9946	$13,289	$13,289
	Phys. fees	$5877	$6596	$7088	$7714	$8571	$2693
	Post-acute	$1110	$1298	$1433	$1615	$2129	$1019

The readmission rates were 6% for PHR, 6% for PKR, 13% for CABG, 7% for LSF, and 20% for colectomy. Within this context, payment variation was observed to be high, with $13,289 and $17,257 for CABG and colectomy, respectively.

## Discussion

Bundling initiatives have been embraced by many health systems since the rollout of the CMS bundled-payment model in 2011. Studies of cost and quality related to bundling have focused mainly on Medicare and Medicaid populations, due to the relative accessibility of the large CMS database. While several studies have included patients of working-age populations, these usually focus on one facility or one set of procedures. To our knowledge, this study presents the largest population of working-age adults yet investigated, and is one of the few to do so across multiple types of procedures. Therefore, this study represents an important contribution to the literature, and the findings are expected to be relevant to other systems wishing to insure working-age adults by means of bundled payment.

We observed substantial variation in price standardized, bundled TRICARE payments for all five procedures. Case-mix adjustment for patient demographics, patient acuity, and the resource use intensity of their procedures modestly reduced, but did not eliminate, the variation in payments between the lowest and highest HRR quintiles. Across each category, index hospitalization payments were major drivers of variation. Therefore, it is possible that differences in clinical care contribute to the variation in index hospitalization payments in this study as well, through factors such as length of stay and outlier payments. Healthcare professional fees were relatively high, but associated with only moderate inter quintile variation between the HRRs. Readmissions were associated with the highest inter quintile variation, high to low, across all surgery types but the rarity of these events mean it is difficult to interpret this as arising from differences in efficiency across the HRRs. Post-acute care payments were relatively small overall and minimally variable across the surgery types and HRRs. A recent TRICARE study which included all of our surgical procedures showed that the average age of those receiving purchased care was 52 and over 88% had a Charlson comorbidity score of 1 or less [17]. It is entirely plausible that the low comorbidity burden, related to the age of beneficiaries, is contributing to the low readmission rate and low utilization of post-acute care in this group.

### Comparisons with variation in Medicare payments

Previous analyses conducted using Medicare claims data demonstrated substantial hospital level variation in price standardized [2] and case mix adjusted [7, 29], total payments for similar surgical procedures types to those used in our study. Our approach focused on Hospital Referral Region variation rather than hospital level variation. Despite this, we found similar patterns of variation for total payments across the HRRs for our surgical procedures of interest as was observed at the hospital level within Medicare claims analyses [6, 7, 29]. Given the methodological differences, a direct comparison is not appropriate, but the similarity of our findings with previous Medicare analyses broadly corroborate our findings and suggest variation in total payments for procedural care between the two populations is fairly similar.

With respect to the variation in payments for the different elements of care, a direct comparison with similar analyses in Medicare is precluded due to the differences in hospital and HRR level approaches. However, looking at general trends we were able to deduce some important differences and similarities between the Medicare population and our study population. This information is needed to determine whether the extent of variation in TRICARE, and thus the rationale for bundling, is similar to the prior state of Medicare before CMS implemented bundling in that system.

The first major difference was in post-acute care expenditure, which was substantially lower and less variable in our study population than previously reported in similar Medicare analyses [6, 7, 29]. This is not due to differences in TRICARE coverage rules since they follow the Medicare coverage rules. As previously alluded to, low rates of utilization within TRICARE purchased care beneficiaries are potentially due to the low comorbidity burden seen in these patients. This also likely contributes to the difference in all-cause readmission rates between the TRICARE (7.7%) and Medicare (19%, 2007 to 2011) [30] populations under study.

The second major difference is in the index hospitalization variation, which appears higher in our population than in previous, similarly-conducted analyses in Medicare claims data [7, 29]. There are three potential reasons for this. First, we formed quintiles across HRRs for each element of care rather than defining a single set of quintiles based on the ranking of total payments. This may provide a more accurate reflection of national level variation in index hospitalization payments, but conceivably makes it look more varied than if the latter method is followed. However, despite price standardization, case mix adjustment, and allowing for DRG heterogeneity, there are clear, national-level differences in payments made to different hospitals for the surgical care of similar patients. For reasons previously described, this is not as evident in the Medicare analyses due to methodological differences, but it still does exist to some extent. Second, the use of HRRs as the unit of variation combined with low numbers of observations in some cases may amplify the observed variation. Third, there may be subtle, proprietary differences in the calculations used by TRICARE vs. Medicare, which would not be reflected in the data set.

### Feasibility

Mandated in the National Defense Authorization Act of 2016, section 726 to test value based purchasing, the Military Health System is overseeing a Value Based Reimbursement Demonstration Project. Thus, additional insight into the feasibility of applying bundled payment to TRICARE purchased care high frequency, high cost surgical services will be informed by the on-going pilot study taking place in Tampa, Florida from May2016-December 2019 [9]. Lessons learned from the demonstration project combined with findings from this current project may be used to shape the future of value-based purchasing for TRICARE.

### Limitations

This study has several limitations. First, as these findings are dependent on TRICARE claims, care sought in non-TRICARE settings or self-paid would not be captured in the data set. However, the highest-cost factors, such as transfer to a skilled nursing facility, are unlikely to be covered at lower patient cost elsewhere. Therefore, we are confident that these findings are generally representative of post-acute care expenditure. Second, the analysis of administrative data may not capture the nuances of the patient’s premorbid physical condition or of their treatment during their clinical care pathways. Inaccurate or “catch all” coding is a known limitation of administrative databases, and has been described in previous papers discussing TRICARE [31– 33]. Similarly, physician services could not be separated confidently into inpatient and outpatient care, and so were grouped independently. Finally, as previously discussed, the compiling of data at the HRR level instead of the hospital level limits the analysis of facility-based cost drivers. This represents a significant opportunity for further study.

## Conclusions

The national level variation in adjusted total payments suggests that patterns of utilization associated with surgical care do vary across the country in adults under 65. However, when payments for the separate elements of care are considered, the patterns in TRICARE data are different from that observed in Medicare data, with hospital related payments representing the main source of variation in expenditure. Therefore, bundled payment plans that cover care for working-age adults are likely to find their greatest cost savings at the hospitalization stage, rather than the post-acute care stage of treatment.

## Supplementary information

**Additional file 1.** Episode Selection.

**Additional file 2.** Defining Payment Buckets.

**Additional file 3.** Price Standardization of Payments.

**Additional file 4.** Case Mix Adjustment.

## Data Availability

The data that support the findings of this study are available from the Defense Health Agency but restrictions may apply to the availability of these data, which were used under a data sharing agreement for the current study, and so are not publicly available. Data are however available from the authors upon reasonable request and with permission of the Defense Health Agency. Information about the Military Health System Data Repository is available at https://www.health.mil/Military-Health-Topics/Technology/Clinical-Support/Military-Health-System-Data-Repository

## Abbreviations

CABG: Coronary artery bypass grafting
CMS: Centers for medicare and medicaid services
DRG: Diagnosis related group
HRR: Hospital referral regions
LSF: Lumbar spinal fusion
MHS: Military health system
PHR: Primary elective hip replacement
PKR: Primary elective knee replacement
