# Supplementary material for: Variation in expenditure for common, high cost surgical procedures in a working age population: implications for reimbursement reform
Source: BMC Health Serv Res. 2019 Nov 21;19:877. doi: 10.1186/s12913-019-4729-2 (PMC6873455; doi:10.1186/s12913-019-4729-2)
Supplement: Supplementary file 1 — Additional file 1. Episode Selection. [file 12913_2019_4729_MOESM1_ESM.docx]

Additional File 1. Episode selection

In addition to the episode selection outlined in the method section using ICD 9 procedural codes (see flow sheet I appendix 1) we took the additional step of applying CMS models for bundled payment models to our groupings. This was done to improve homogeneity within our surgical groupings and to focus on variation thought by CMS to be targetable through bundled payments. As such we filtered our episodes to exclude those which were not solely linked to a DRG within a surgery specific CMS bundled payment model. For example in the case of CABG we applied the DRG list for the CMS BPCI CABG model 2 to our episodes identifies by ICD 9 Procedural codes. The following shows which models and DRG (Diagnosis Related Group) codes were used for each surgical grouping.

| **Elective Surgery type** | **CMS Bundled payment model** | **DRG list** |
| --- | --- | --- |
| CABG | Coronary Artery Bypass Surgery, BPCI, model 2 | 231,232,233,234,235,236 |
| Total knee replacement | Comprehensive joint replacement CJR model | 469,470 |
| Total hip replacement | Comprehensive joint replacement CJR model | 469,470 |
| Colectomy | Major bowel, BPCI, model 2 | 329,330,331 |
| Lumbar spinal fusion | Spinal fusion (non-cervical), BPCI, Model 2 | 459,460 |
