# Supplementary material for: Variation in expenditure for common, high cost surgical procedures in a working age population: implications for reimbursement reform
Source: BMC Health Serv Res. 2019 Nov 21;19:877. doi: 10.1186/s12913-019-4729-2 (PMC6873455; doi:10.1186/s12913-019-4729-2)
Supplement: Supplementary file 2 — Additional file 2. Defining Payment Buckets. [file 12913_2019_4729_MOESM2_ESM.docx]

Additional File 2. Defining Payment Buckets

The following were the definitions used to define the payment buckets:

**Index hospitalization payment:**

‘Total amount paid by TRICARE for the index procedure and associated inpatient stay on behalf of an individual, excluding professional fees.’

**Readmission payments:**

‘Total amount paid by TRICARE for any subsequent inpatient treatment linked to the index procedure occurring within the 90-day period since the discharge from the index procedure, excluding professional fees.’

**Professional payments:**

‘Total amount paid by TRICARE on behalf of any patient for care provided by any medical professional during the index procedure and the 90-day period since the discharge from the index procedure, including inpatient or outpatient setting.’

**Post-acute care payments:**

‘Total amount paid by TRICARE to facilities on behalf of a patient in the days following discharge from the index procedure but within the 90-day time period, excluding professional fees. This will include payments to Rehabilitation hospitals, Skilled Nursing Facilities, Long Term Care Facilities, Home Health Nursing Homes, Hospices and Outpatient facility fees’

DRG and CPT (Common Procedural Codes) codes were temporally related to the index procedures. TRICARE field ‘insttype’ was used to identify the setting in which DRG related care was undertaken- inpatient or outpatient. TRICARE field ‘provspec’ was used to differentiate professional fees and facility fees for CPT codes.
