# Supplementary material for: Variation in expenditure for common, high cost surgical procedures in a working age population: implications for reimbursement reform
Source: BMC Health Serv Res. 2019 Nov 21;19:877. doi: 10.1186/s12913-019-4729-2 (PMC6873455; doi:10.1186/s12913-019-4729-2)
Supplement: Supplementary file 3 — Additional file 3. Price Standardization of Payments. [file 12913_2019_4729_MOESM3_ESM.docx]

Additional File 3. Price Standardization of Payments

All payments were standardised following methods described by researchers with the Dartmouth Atlas ^13,1^. TRICARE claims data is not structured in the same way as Medicare data and costs can only be linked to DRG and CPT codes.

DRG payment standardisation followed the formula included at the end of this section. This was used for all DRG payments which were associated with the index hospitalisation, readmissions and post-acute care in the form of rehabilitation DRGs. The TRICARE specific DRG payment, calculated by multiplying the 2013 Medicare DRG base weight by the 2013 Medicare DRG weighting, was subtracted from the total amount paid by TRCIARE for the hospital in patient stay. The remainder in excess of the DRG payment was then divided by the 2013 wage index to produce a standardised figure.

CPT code standardisation according to Dartmouth methods involves identifying hospital level characteristics such as payments specific to medical education and indemnity. Since we were unable to identify specific hospitals we pragmatically adopted a top down approach and substituted the average for the whole population for each specific CPT code when one was present.

*Formula for price standardisation of DRGs*

Price Standardised payment= DRG*P + (Paid-(DRG*P))/WI

DRG= 2013 Medicare base rate

P= 2013 Medicare DRG weightings

Paid= Field indicating total payment made by TRICARE for inpatient care

WI= Wage index for 2013
