# Supplementary material for: Variation in expenditure for common, high cost surgical procedures in a working age population: implications for reimbursement reform
Source: BMC Health Serv Res. 2019 Nov 21;19:877. doi: 10.1186/s12913-019-4729-2 (PMC6873455; doi:10.1186/s12913-019-4729-2)
Supplement: Supplementary file 4 — Additional file 4. Case Mix Adjustment. [file 12913_2019_4729_MOESM4_ESM.docx]

Additional File 4. Case mix adjustment

All payments for all episodes were adjusted using the following patient specific characteristics.

- Age
- Sex
- Race
- Rank or sponsors rank if dependent
- Charleson comorbidity index
- Total prior six month, to admission, healthcare expenditure

**Coronary artery bypass graft (CABG)**

Only primary elective bypass graft surgery episodes were considered, all episodes were this was combined with another surgery were excluded from analysis. Adjustment was made to allow for the number of vessels bypassed during the procedure to reflect degree of severity of the pathology and the resource implications of the procedure.

**Colectomy**

Case mix adjustment was made to account for partial or total colectomy and if the aetiology was cancerous or non-cancerous to reflect the resource implications during the initial procedure and the post-operative care.

**Lumbar spinal fusion (LSF)**

Case mix adjustment was made to allow for single or multiple level fusions to reflect differences in resource utilisation.

**Total knee replacement (PKR)**

Only total joint arthroplasties were considered. No specific surgical adjustments were made.

**Total hip replacement (PHR)**

Only total joint arthroplasties were considered. No specific surgical adjustments were made.
